# Rare Presentation of FLT3-ITD-Positive Acute Myeloid Leukemia With Monocytic Differentiation: A Case Report

**DOI:** 10.7759/cureus.32988

**Published:** 2022-12-27

**Authors:** Samaher S Hazzazi, Abdullah W Bormah, Hamzah H Alsabban, Adel Al-Marzouki, Salem Bahashawan, Yara Daous

**Affiliations:** 1 Medicine, King Abdulaziz University Faculty of Medicine, Jeddah, SAU; 2 Hematology, King Abdulaziz University Faculty of Medicine, Jeddah, SAU; 3 Hematopathology, King Abdulaziz University Faculty of Medicine, Jeddah, SAU

**Keywords:** hand amputation, rare case report, thromboses, flt3-itd, acute myeloid leukemia (aml)

## Abstract

Acute myeloid leukemia (AML) is a hematological malignancy that affects adults and has various presenting symptoms, the most common being shortness of breath, bleeding, and infection. Thrombosis is also believed to be a rare presenting symptom of AML; however, information about the association between AML and thrombosis is scarce. Here, we report the case of a 27-year-old female who presented with extensive coagulation disturbances leading to various thromboembolic complications (including multiple strokes and renal and splenic infarcts) and was eventually diagnosed with AML. Owing to the patient’s functional status at diagnosis, chemotherapy induction was withheld, and close observation along with supportive treatment was initiated. The findings, in this case, provide useful information on the presentation of such unusual cases, so we aim to enrich and contribute to medical evolution.

## Introduction

Acute myeloid leukemia (AML) is characterized by immature hematopoietic cell proliferation in the bone marrow [1]. It is the most prevalent acute leukemia in adults that affects the myeloid lineage [2]. Although venous thromboembolism (VTE) is a common side effect in cancer patients, data regarding the incidence of thromboembolism in patients with AML are insufficient [3]. Shortness of breath, infection, and bleeding are among the most common symptoms exhibited by patients with AML; however, thrombosis is still considered a rare presenting symptom [1]. The pretreatment karyotype and molecular biomarkers are important criteria for AML risk stratification and prognosis [2]. Particularly, the FMS-like tyrosine kinase 3-internal tandem duplication (FLT3-ITD) mutation is associated with poor prognosis [2].

## Case presentation

A 27-year-old Nigerian female presented to the Emergency Department complaining of left flank pain, fever, and loss of appetite for three weeks. The patient had returned from her home country three weeks prior to the presentation. Eleven days after arrival, she developed a productive cough with whitish sputum and sneezing. As her illness progressed, she developed fever (reaching 38.5°C), chills, recurrent episodes of vomiting, and decreased appetite. Three days before admission, all her baseline symptoms worsened, in addition to developing reduced responsiveness, which indicated an altered mental status. She presented with tachycardia (heart rate, 120 beats per minute), tachypnea (respiratory rate, 35 breaths per minute), high-grade fever (38.8 °C), and hypotension (blood pressure, 82/49 mmHg); however, oxygen saturation was maintained at >95% on ambient air. Physical examination revealed a dehydrated pale patient with a fatigued appearance; however, she was still conscious, alert, and oriented. A state of shock was declared, and multiple boluses of fluid and albumin were administered to the patient until her blood pressure was elevated to within the normal range. Broad-spectrum antibiotics were started after resuscitation. Further examination revealed bilateral equal air entry into the lungs with basal crepitations, hepatosplenomegaly, and significant left upper quadrant abdominal tenderness upon palpation.

Preliminary laboratory tests revealed leukopenia (white blood cell count, 3.84x10^3/mm3, reference range: 4.5-11.5), thrombocytopenia (platelet count, 58,000, reference range: 150,000-450,000) in addition to normocytic anemia (hemoglobin level, 7.4 g/dL, reference range: 12.0-15.0; mean corpuscular volume, 84 fL, reference range 80-94), hypokalemia (2.9 mmol/L, reference range: 3.5-5.1), and hyponatremia (131 mmol/L, reference range: 136-145). The coagulation profile was suggestive of an ongoing coagulopathy, with a prolonged international normalized ratio (2.85, reference range: 0.89-1.18), prolonged prothrombin time (36 s, reference range: 11.7-15.3), prolonged activated partial thromboplastin time (54 s, reference range, 28.9-38.1), a reduced fibrinogen level (<40 mg/dL, reference range: 200-400), and an elevated D-dimer level (20 mg/L, reference range: 0-0.5). 

Liver enzymes exhibited hepatocellular damage (aspartate aminotransferase, 295 U/L, reference range: <34; alanine transaminase, 409 U/L, reference range: 10-49), with subsequent development of cholestasis (alkaline phosphatase, 231 U/L, reference range: 46-116; gamma-glutamyl transferase, 398 U/L, reference range: <38). Total bilirubin was slightly elevated (36 umol/L, reference range: 5-21) with normal albumin (44 g/L, reference range: 40.2-47.6). Antiphospholipid antibody testing was negative, and the blood film comment revealed abnormal mononuclear/immature cells. Venous blood gas evaluation at presentation was suggestive of respiratory alkalosis.

**Figure 1:**
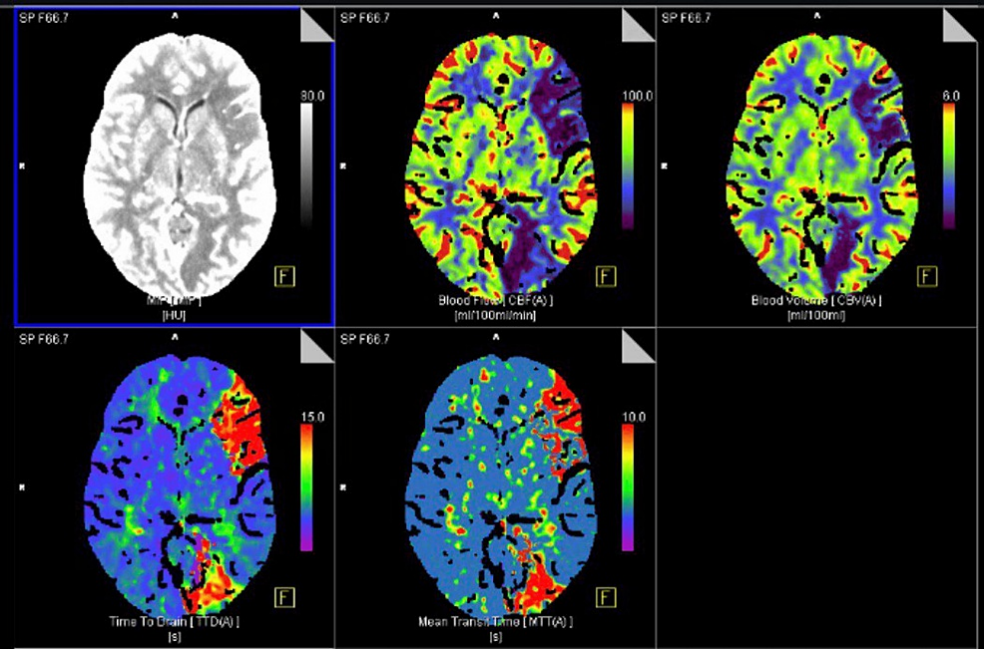
Computed tomography perfusion shows ischemic insult leading to hypo perfusion of the left frontal and occipital regions.

**Figure 2:**
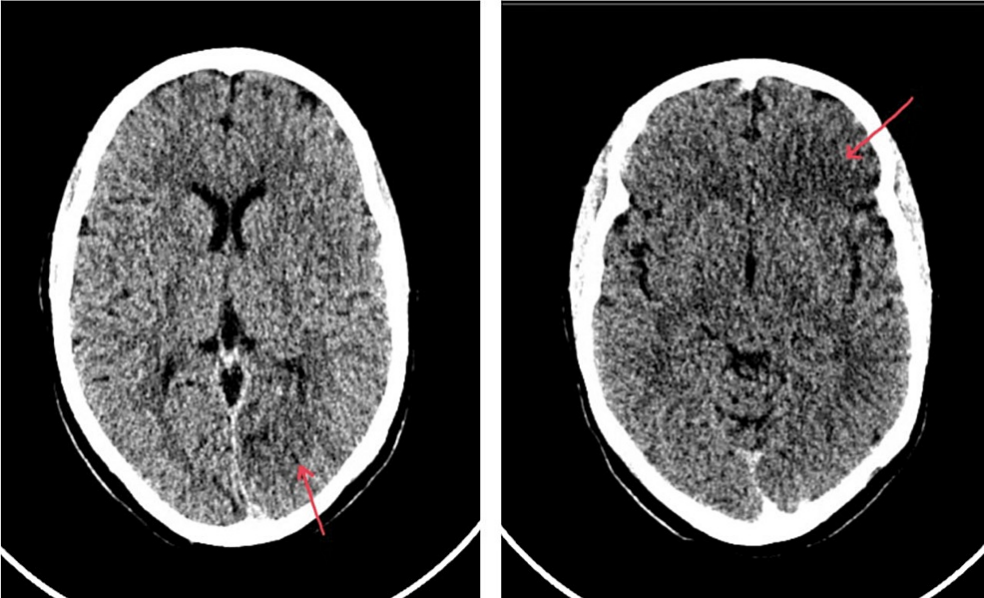
Computed tomography of the brain without contrast shows distal infarctions in the frontal and occipital regions.

During monitoring after resuscitation, the patient suddenly developed right-sided paralysis and aphasia, which necessitated the activation of the stroke protocol. Computed tomography (CT) of the brain revealed a left fronto-occipital distal middle cerebral artery stroke (Figures 1, 2), and as per the neurologist’s assessment, she was not a candidate for thrombolysis or thrombectomy. Magnetic resonance imaging (MRI) of the brain was done 12 days after admission to rule out other intracranial causes of her altered mental status and it revealed the hemorrhagic transformation of the infarcts. 

The patient’s condition at the time was critical; therefore, she was shifted to the intensive care unit (ICU) for closer monitoring and ongoing management, where she stayed for 15 days. She required intubation due to a drop in the Glasgow Coma Scale score, and a central line was inserted due to persistent hypotension. Dexamethasone was added to the treatment as well. Unfortunately, one day after admission, after inserting a radial line, her right hand started to show dark discoloration and mottling. Vascular surgeons diagnosed another thrombotic event and suggested that she was not a candidate for revascularization due to the occlusion occurring in the distal digital arteries. A clear line of demarcation was awaited, and the patient underwent an above-the-wrist amputation of the right hand by orthopedic surgeons on day 26 after admission. 

**Figure 3:**
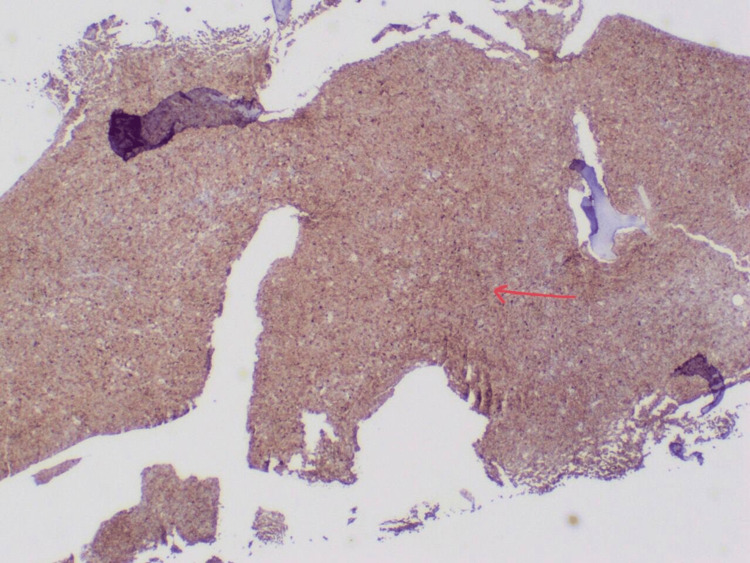
Bone marrow biopsy strongly positive for CD45.

During her hospital stay, AML was suspected based on abnormal immature cells in the peripheral blood smear. Bone marrow biopsy revealed blast cells strongly and diffusely positive for CD45 (Figure 3), CD4, CD86, and CD163, and hypercellular marrow with sheets of blasts comprising 99% of marrow cellularity and trilineage hematopoiesis was markedly diminished, which was also indicative of leukemia.

Molecular and genomic testing of bone marrow specimens indicated the presence of FLT3-ITD mutation but was negative for nucleophosmin-1 (NPM1), IDH1(R132), and IDH2(R172). Chromosomal analysis revealed a normal karyotype. 

**Figure 4:**
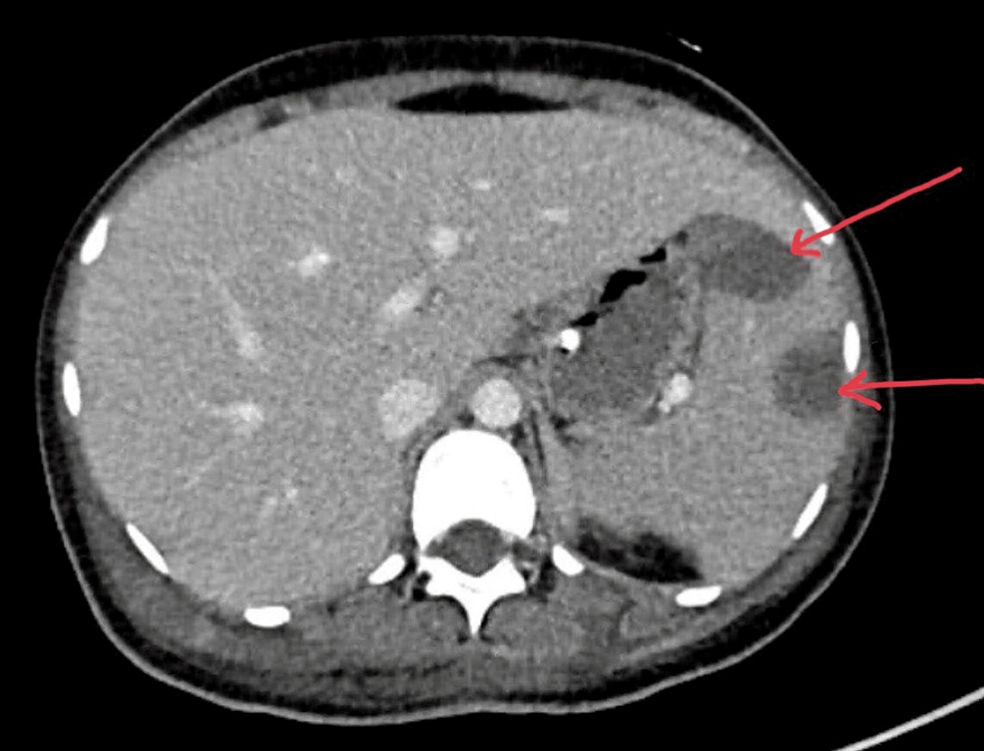
Axial view of CT abdomen shows two large wedge-shaped hypodense lesions representing infarcts.

**Figure 5:**
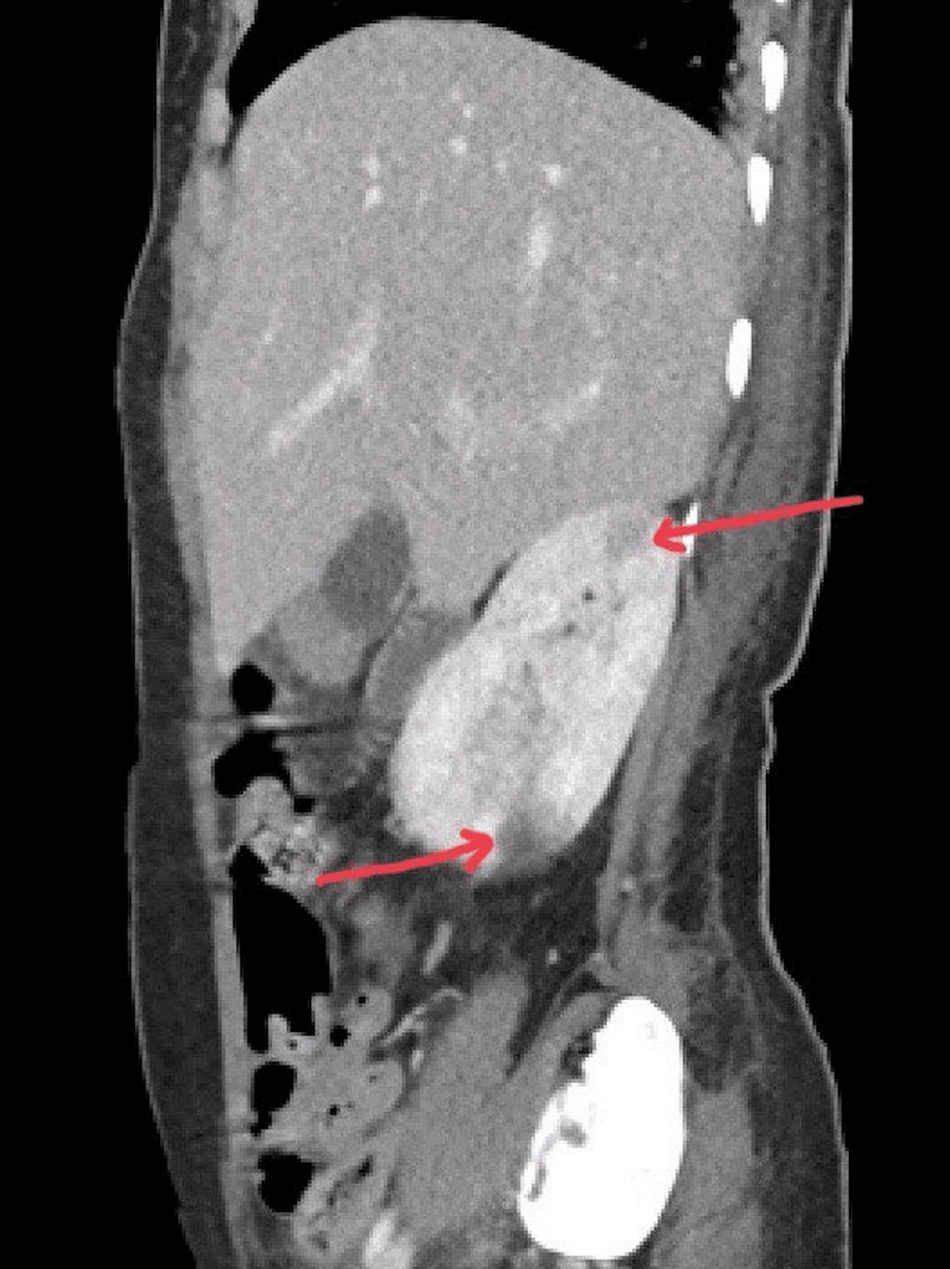
Sagittal view of CT abdomen shows multiple hypodense lesions likely representing infarcts.

Airspace opacification/infiltration was noted in the lower lobe of the left lung. The initial abdominal CT revealed hepatosplenomegaly, a 2.2-cm liver hemangioma, multiple large wedge-shaped splenic infarcts (Figure 4), and enlarged bilateral adrenal glands. Follow-up CT 10 days after admission showed interval development of new splenic and bilateral renal infarcts (Figure 5) and suspicious linear rectal hyperdensity with pooling suggestive of active intraluminal bleeding.

**Figure 6:**
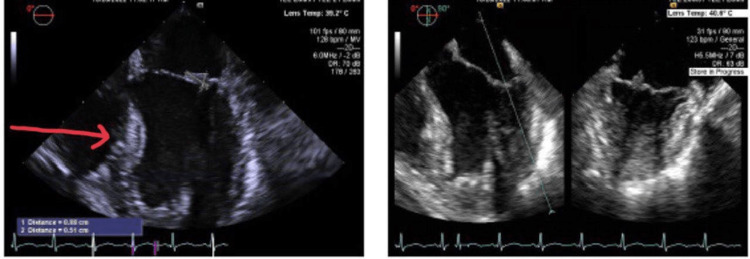
Transesophageal echocardiogram shows mobile pedunculated vegetation on the mitral valve.

Transthoracic echocardiography was performed twice during the hospitalization and was inconclusive for features of endocarditis on both occasions, but showed mildly reduced ejection fraction (45-50% by visual estimation) and mild pericardial effusion. Follow-up transesophageal echocardiogram (Figure 6) revealed pedunculated mobile vegetation on the mitral valve measuring 0.8 x 0.5 cm, which was later diagnosed as non-infectious endocarditis (marantic endocarditis).

**Figure 7:**
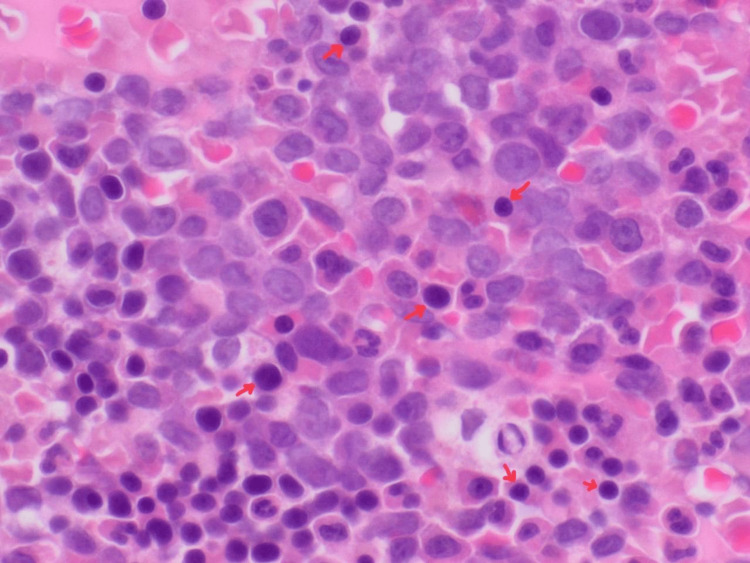
Repeat bone marrow biopsy shows marrow regeneration and the presence of adequate erythroid precursors.

Based on the patient’s Eastern Cooperative Oncology Group (ECOG) Performance Status Scale and Karnofsky Performance Scale scores (4 and 20 points, respectively), the patient was unlikely to be able to tolerate chemotherapy, and induction of chemotherapy was decided to be withheld. Continuous monitoring and supportive care were initiated while waiting for her clinical status to improve, and she started recovering her functional status, developed bone marrow regeneration and adequate erythroid precursors (Figure 7) along with increased megakaryocytes based on a repeat biopsy without initiating chemotherapy. The patient is now clinically stable apart from residual weakness after the stroke. The plan is to monitor her clinically in regard to functional status and neurological deficits, do weekly CBC and coagulation profiling, as well as an echocardiogram annually. 

## Discussion

Malignancy is linked to a "hypercoagulable" condition and an increased risk of thrombotic and hemorrhagic complications. Even though disseminated intravascular coagulation in AML has been reported, it is widely assumed that VTE in AML is uncommon [4]. Overall, there is little information available on the possibility of thrombosis in those cases, especially thrombosis of large vessels, which is considered quite unusual [4,5].

We reported the case of a young patient with FLT3-ITD-positive AML with a rare presentation and outcome. The patient presented with various thromboembolic events, including multiple ischemic strokes with hemorrhagic transformation, renal and splenic infarcts, rectal bleeding, non-infectious “marantic” endocarditis, and hand gangrene that led to amputation.

In a study of 379 patients with acute leukemia and thrombosis conducted in Italy, only 3.2% of the included population manifested clinical thrombosis at presentation; and as few as 1.3% had ischemic strokes (a total of five patients out of 379), of which two were fatal [5]. Similarly, our patient demonstrated an unusual acute ischemic stroke upon presentation and subsequent multiple thromboses.

According to another study that included 5394 patients with AML, it was reported that advanced age, female sex, number of chronic comorbid conditions, and a central venous catheter presence are all predictive factors for VTE [6]. None of the above-mentioned risk factors explains our case, except for sex in addition to age. In particular, the age of our patient is 27, which has been reported to be of borderline significance in terms of VTE development. 

Undoubtedly, patients with leukemia and solid malignancies frequently exhibit abnormalities in blood coagulation tests, even in the absence of clinical signs of thromboembolism or hemorrhage [4]. Nonetheless, our patient demonstrated both clinical and laboratory abnormalities suggestive of disseminated intravascular coagulation including markedly elevated D-dimer levels (20 mg/L), prolonged clotting time (prothrombin time and activated partial thromboplastin time), reduced platelet counts, and decreased fibrinogen concentration.

The FLT3 gene mutation is a very prevalent somatic mutation in AML and is found in 25-45% of patients [7]. ITD is the most prevalent FLT3 mutation observed in AML patients. FLT3-ITD is a substantial mutation that signifies a severe leukemic issue, grants poor prognosis, and has a serious negative effect on AML management [8,9]. Surprisingly, although our patient had an FLT3-ITD mutation, she was clinically stable and recovered her functional and mental status without the induction of chemotherapeutic agents. A follow-up bone-marrow biopsy revealed recovery of bone marrow components, and the peripheral blood smear started to normalize. This phenomenon is rare and scarcely reported in the literature.

Limitations

Limitations of this case include the inability to initiate chemotherapy, which makes it challenging to determine the impact of chemotherapy on the outcome of such cases. Also, FLT3-ITD molecular aberrations have a statistically significantly worse prognosis in patients with AML [2]. However, we were unable to conduct further molecular studies and the variable allele frequency (VAF) was not reported due to limited resources, and we were unable to determine whether the patient had any additional molecular abnormalities that could have contributed to her catastrophic presentation.

## Conclusions

AML can present in various ways as shown in our case. Some of those presentations might provide difficulty in establishing a correct and quick diagnosis so as to initiate curative intended treatment. Thus, performing all the necessary and extensive cytogenetic workup and having a high index of suspicion is an excellent pathway for risk stratification and possible cures for such an aggressive debilitating disease.

FLT3-ITD gene mutation conveys a poorer prognosis, but our case demonstrated improvement in functional status and restoration of bone marrow and blood components with supportive treatment and without initiation of chemotherapy. Although thrombosis presents in a minority of AML patients, physicians should always be cautious when dealing with suspected cases to prevent irreversible damage to vital organs and subsequently affect the quality of life for those patients.

## References

[1] Greenfeld SM, Tadmor T (2021). 'Catastrophic' thrombosis in a young patient with acute myeloid leukemia presenting early in the COVID-19 pandemic - a case report. In Vivo.

[2] Padmakumar D, Chandraprabha VR, Gopinath P (2021). A concise review on the molecular genetics of acute myeloid leukemia. Leuk Res.

[3] Libourel EJ, Klerk CP, van Norden Y (2016). Disseminated intravascular coagulation at diagnosis is a strong predictor for thrombosis in acute myeloid leukemia. Blood.

[4] Barbui T, Falanga A (2001). Disseminated intravascular coagulation in acute leukemia. Semin Thromb Hemost.

[5] De Stefano V, Sorà F, Rossi E (2005). The risk of thrombosis in patients with acute leukemia: occurrence of thrombosis at diagnosis and during treatment. J Thromb Haemost.

[6] Ku GH, White RH, Chew HK, Harvey DJ, Zhou H, Wun T (2009). Venous thromboembolism in patients with acute leukemia: incidence, risk factors, and effect on survival. Blood.

[7] Tyner JW, Erickson H, Deininger MW (2009). High-throughput sequencing screen reveals novel, transforming RAS mutations in myeloid leukemia patients. Blood.

[8] Bacher U, Haferlach C, Kern W, Haferlach T, Schnittger S (2008). Prognostic relevance of FLT3-TKD mutations in AML: the combination matters--an analysis of 3082 patients. Blood.

[9] Auewarakul CU, Sritana N, Limwongse C, Thongnoppakhun W, Yenchitsomanus PT (2005). Mutations of the FLT3 gene in adult acute myeloid leukemia: determination of incidence and identification of a novel mutation in a Thai population. Cancer Genet Cytogenet.

